# Topological hybrid silicon microlasers

**DOI:** 10.1038/s41467-018-03434-2

**Published:** 2018-03-07

**Authors:** Han Zhao, Pei Miao, Mohammad H. Teimourpour, Simon Malzard, Ramy El-Ganainy, Henning Schomerus, Liang Feng

**Affiliations:** 1 Department of Electrical and Systems Engineering, University of Pennsylvania, Philadelphia, PA 19104 USA; 2 Department of Materials Science and Engineering, University of Pennsylvania, Philadelphia, PA 19104 USA; 3 Department of Electrical Engineering, The State University of New York at Buffalo, Buffalo, NY 14260 USA; 4 Department of Physics and Henes Center for Quantum Phenomena, Michigan Technological University, Houghton, MI 49931 USA; 5 Department of Physics, Lancaster University, Lancaster, LA1 4YB UK

## Abstract

Topological physics provides a robust framework for strategically controlling wave confinement and propagation dynamics. However, current implementations have been restricted to the limited design parameter space defined by passive topological structures. Active systems provide a more general framework where different fundamental symmetry paradigms, such as those arising from non-Hermiticity and nonlinear interaction, can generate a new landscape for topological physics and its applications. Here, we bridge this gap and present an experimental investigation of an active topological photonic system, demonstrating a topological hybrid silicon microlaser array respecting the charge-conjugation symmetry. The created new symmetry features favour the lasing of a protected zero mode, where robust single-mode laser action in the desired state prevails even with intentionally introduced perturbations. The demonstrated microlaser is hybrid implemented on a silicon-on-insulator substrate, and is thereby readily suitable for integrated silicon photonics with applications in optical communication and computing.

## Abstract (web summary)

Topological effects, first observed in condensed matter physics, are now also studied in optical systems, extending the scope to active topological devices. Here, Zhao et al. combine topological physics with non-Hermitian photonics, demonstrating a topological microlaser on a silicon platform.

## Introduction

The discovery of topological band theory has ushered in a new era in condensed matter physics, providing intriguing insights into the world of low-dimensional quantum systems featuring, e.g., the quantum Hall effect and quasiparticles with fractional statistics, and paving the way to engineer new states of matter, such as topological insulators and superconductors^1^. Inspired by this groundbreaking work, topological mechanisms of optical mode formation have been proposed^2^. The subsequent investigations of passive topological photonic systems have facilitated unidirectional transport channels in photonic crystals^3^, optical cavity arrays^4,5^, as well as helical waveguide lattices^6^, and the demonstration of robust topological defect states in metamaterial arrangements and dielectric resonators^7,8^, among a host of other intriguing effects^9,10^ that even extend to acoustic systems^11^. However, these pioneering studies have been limited in scope, exploring only a small subset of the full design parameter space^12,13^.

Active optical systems involving feedback mechanisms provide a much wider arena. Most recently, considerable effort has been made to transplant the topological notions into lasing systems^14–16^, in which topological robustness collides with other physical considerations, posing diverse unexplored fundamental questions about the interplay between topological features, non-Hermitian physics^17–24^, and the break-down of the superposition principle. The answers to these questions transform our understanding of topological robustness by revealing unique connections between topology and other types of fundamental symmetries arising from non-Hermiticity (naturally pertinent to active systems), thus opening the door for improving robust optical device functionality, a key incentive in the research of integrated photonics over the past few decades. This new paradigm dictates a fresh look at the basic notion of topological protection in order to take into account the expanded design parameters space^25–31^, and to establish a connection between topological physics and various separate activities on non-Hermitian photonic systems^32–34^.

Here, we experimentally explore the utility of topological concepts to active systems and demonstrate an on-chip hybrid silicon microlaser whose mode competition naturally favours robust laser action arising from a topological defect. Different from a recent breakthrough demonstration of topological edge-mode lasing where the edge state is selectively excited^35^, our microlaser is based on the strategic combination of non-Hermitian and topological symmetries, supporting arbitrary pumping strategies (either uniform or selective pumping).

## Results

### Topological microlaser array on a hybrid silicon platform

**Fig. 1:**
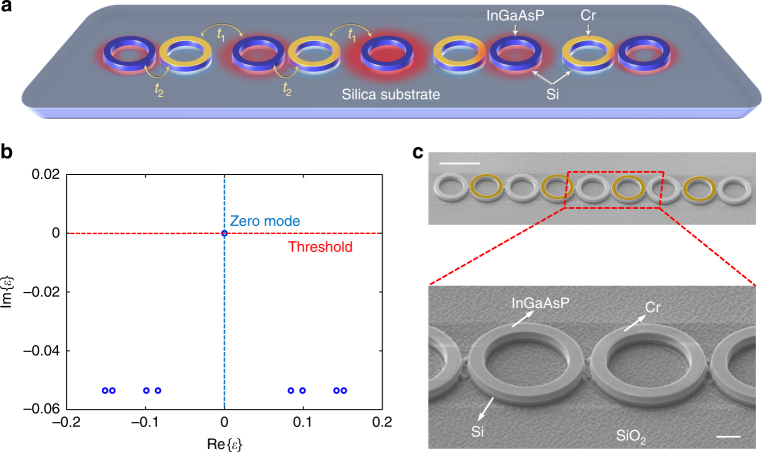
Topological hybrid silicon microlaser. **a** Schematic of a topological laser array made of nine microring resonators with alternating weak (*t*_1_) and strong (*t*_2_) couplings, emulating an SSH model. A layer of 10-nm Cr (shown in yellow) is deposited on top of every second element to introduce distributed gain and loss. The red halos represent the intensity profile of the oscillating zero mode that resides at the central site and decays exponentially away from the centre, with zero intensity in every second element. **b** Spectral features of the topological laser array, highlighting the lasing selectivity of the topological zero mode. Due to the charge-conjugation symmetry, the spectrum is symmetric around the imaginary axis. The blue circles represent the calculated eigenvalues of all the nine supermodes of the laser array. **c** SEM pictures of the fabricated structure consisting of nine rings on a hybrid III–V/silicon platform. Each ring has inner and outer radii of 3.5 and 4.5 µm, respectively, and is made of 500 nm-thick InGaAsP quantum well layers on top of a 220 nm silicon layer grown on a silicon-on-insulator (SOI) substrate. The alternating 200 and 300 nm edge-to-edge separations between adjacent rings realise the weak coupling *t*_1_ = 78 GHz and the strong coupling *t*_2_ = 134 GHz, compositing an estimated Hermitian topological band gap of 112 GHz in the absence of loss. In the top panel of **c**, we highlighted the Cr layer with artificial yellow rings for better visualisation of the arrayed structure, since the Cr layer is only ~10 nm thick and difficult to be clearly seen, especially in pictures of low magnification. Scale bars in **c**: low magnification: 10 µm; high magnification: 2 µm

Our topological laser structure is an array of coupled microring resonators (Fig. 1a), motivated by a non-Hermitian variant^25,26^ of the paradigmatic Su-Schrieffer-Heeger (SSH) model^36,37^, a tight-binding Hamiltonian whose topological features arise from a sequence of alternating couplings and that adapts flexibly to many physical settings^38,39^. The coupling profile is precisely controlled by the separations between adjacent rings in an alternating fashion, which in turn determine the strength of the evanescent wave tunnelling rate. A spacing defect in the centre of the array creates a topological zero mode that decays exponentially away from the defect, and only populates every other resonator. In comparison to the edge mode^35^, our defect mode is well isolated from leakage at the end; furthermore, such spacing defects can be created as desired anywhere within the structure. Spectrally, the topologically protected zero mode resides at the centre of a band gap, where the symmetric features of the passive band structure arise from a chiral symmetry—a symmetry that maps the two symmetric bands onto each other^1^. This symmetry is specifically related to inverting the sign of the couplings^40^, which forces the zero mode onto one “bright” sublattice (here, every second resonator including the defect location)^37^. The distributed gain and loss follows a non-Hermitian charge-conjugation symmetry (a non-Hermitian variant of the chiral symmetry, which also distinguishes the zero mode in terms of its lifetime), leading to a response that robustly discriminates between the topological and non-topological states^26^ as demonstrated by linear passive mode guiding of microwaves^27^ (see Supplementary Note 1 for detailed analysis). Considered in the complex frequency plane, this directly translates into an enhanced gain of the topological zero mode, therefore favoring it over other states throughout the nonlinear mode competition process (Fig. 1b), in which a novel dynamical notion of topological protection persists (see Supplementary Note 2). In our experiment, a hybrid III–V silicon semiconductor platform is chosen to deliver a robust silicon laser for maximising its potential for photonic integrated circuits. Fig. 1c depicts a scanning electron microscope (SEM) picture of the fabricated topological microlaser array, consisting of nine coupled InGaAsP-silicon microring resonators on a silicon-on-insulator (SOI) substrate. As the number of resonators is odd, the zero mode is compatible with the boundary conditions, so that the main effect of the finite system size is the quantisation of the extended states. In order to generate the desired gain/loss distribution, a layer of approximately 10 nm chromium (Cr) is deposited on top of every second resonator using overlay electron beam lithography (EBL) (see Methods). Finally, the gain profile is provided through uniform optical pumping applied from the top (see Methods).

### Characterisation of single-topological-mode lasing

**Fig. 2:**
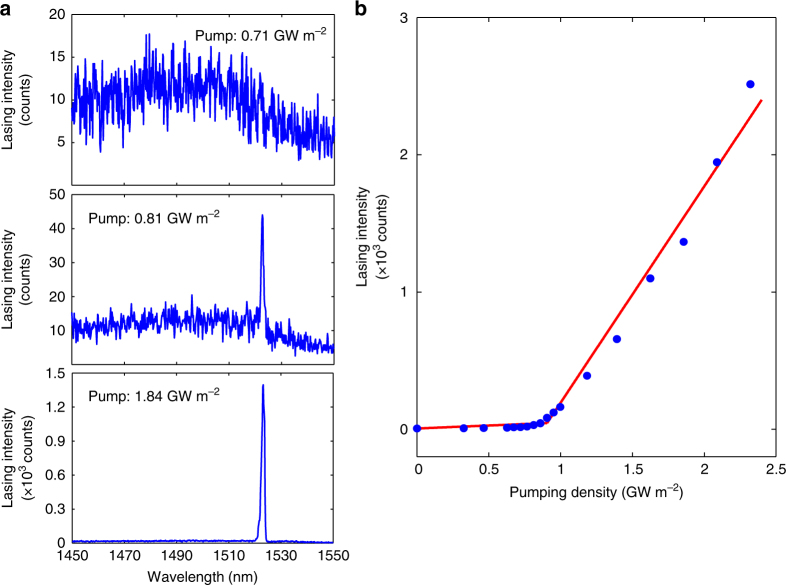
Topological laser action. **a** Lasing spectrum of the structure of the topological hybrid silicon microlaser as a function of the pumping power much below the threshold (top panel), approaching the threshold (middle panel) and well above the threshold (bottom panel). As expected, as the pump power is increased, the output emission experiences a transition from broadband photoluminescence to a single narrow band lasing with an increased extinction ratio. **b** Pump dependence of the laser emission intensity, demonstrating the fingerprint of single-mode lasing: only one threshold with no other kinks in the light–light curve. Blue dots are experimental data and the red lines are linear fits with least squares of the data before and after the lasing threshold, respectively

The spectral properties of the laser action are characterised under different optical pumping levels. While the coupled microring array in principle supports multiple longitudinal modes, only the zero mode can emerge above the lasing threshold due to the introduced topology/non-Hermiticity interplay. The measured spectral evolution of the topological hybrid silicon microlaser clearly manifests a significant spectral narrowing from broadband photoluminescence to amplified spontaneous emission (ASE), and finally to persistent single-mode lasing when well above the lasing threshold (Fig. 2a). Due to the topological robustness associated with the zero mode, the desired single-mode operation is persistent with the resonant peak remaining well isolated around a wavelength of ~1523 nm from ASE to lasing, while the corresponding extinction ratio drastically increases to a value of ~20 dB. Fig. 2b shows the light–light curve, where the pump dependence of the total emitted intensity agrees well with the expectations for single-mode laser action, as it only displays a single threshold without further kinks.

### Large-area single-transverse-mode lasing

**Fig. 3:**
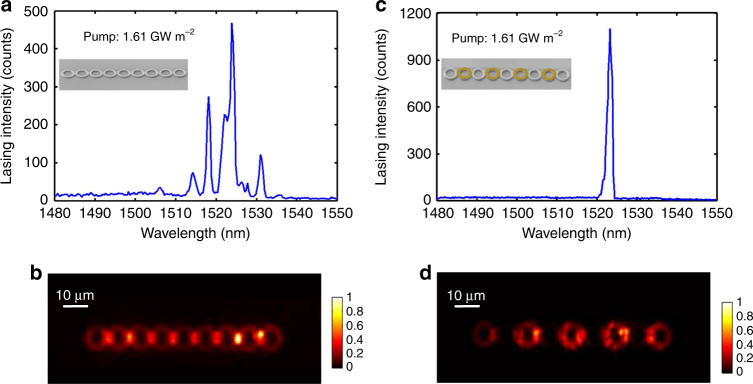
Intrinsic single-supermode topological laser action. **a** Multimode lasing from an identically sized microlaser array as the topological microlaser, but without on-top Cr deposition on every second ring to introduce the distributed gain/loss profile. **b** Measured lasing mode profile of the topological microlaser without on-top Cr deposition. **c** Single-supermode lasing from the topological microlaser under the same pumping condition. **d** Measured lasing mode profile of the topological microlaser with the distributed gain/loss profile. The broad spectrum in **a** is due to mode competition between the transverse and longitudinal modes of the single-ring resonators, each of which forms its own collective state. In **c**, the distributed gain and loss judiciously spoils the quality factors of the strongly hybridised modes, topologically favoring only the zero mode centred around *λ* = 1523 nm

A large overlap between the lasing-mode profile and the gain material is desired in order to achieve high efficiency. In our experiment, therefore, we intentionally design a large-area single-mode laser with the transverse dimension of the hybrid ring being 1 µm wide and 720 nm thick (500 nm InGaAsP and 220 nm silicon). In this regard, while each ring supports several transverse modes, the fundamental transverse mode selected for the zero mode (TM_11_ mode in our work) occupies a much larger area of gain compared with the array of single-transverse-mode rings. In order to confirm the role of topological features in this enriched mode selection process, a control experiment was conducted using an identically sized microlaser array without the designed distributed gain/loss profile. As expected, the hybridisation through couplings of all the transverse and longitudinal modes under the uniform pumping scenario displays a broader emission spectrum with multiple peaks and a reduced peak intensity (Fig. 3a), with the total emission homogeneously distributed over the entire structure (Fig. 3b). In contrast, the zero-mode lasing in the topological array is highly reliable, despite the mode competition in each ring and between the rings (Fig. 3c), which is a direct outcome of the interplay between the topological mode hybridisation and non-Hermiticity (see Supplementary Note 3). The lasing action of the topological zero mode is further validated by the measurement of the spatial lasing mode profile presented in Fig. 3d.

### Robust lasing oscillation against local perturbation

**Fig. 4:**
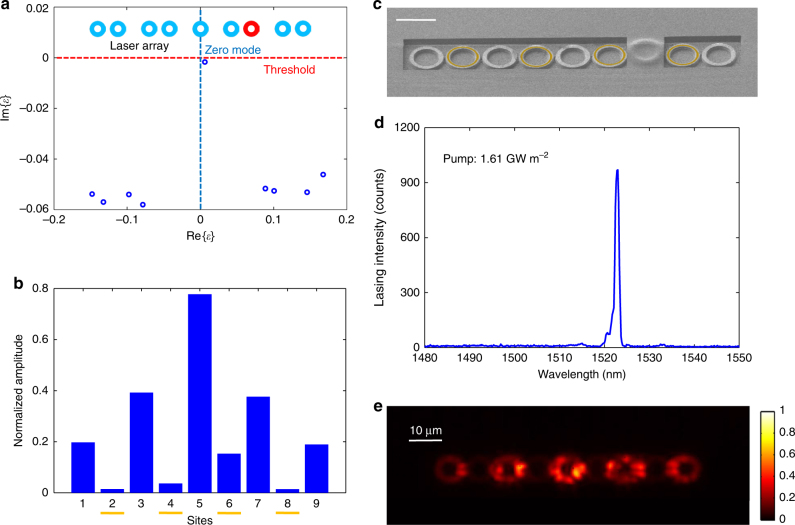
Effect of disorder on the topological microlaser. **a** Numerical calculations for the spectrum of a disordered lattice when an onsite perturbation is introduced to the third site from the right (highlighted in red), as indicated schematically at the top of the same panel. **b** The mode profile of the topological eigenstate, normalised with identity norm calculated from the perturbed tight-binding Hamiltonian, where the general characteristics of the zero mode persist with addition of small light intensities on the previously dark sublattice (labelled in yellow marks). **c** SEM image of the perturbed topological laser array with an on-top thick layer of polymer covering the corresponding ring. Scale bar: 10 µm. **d** Measured emission spectrum of the perturbed laser array showing the maintained single-mode lasing feature against the introduced onsite perturbation. **e** Measured mode profile of the perturbed laser array. Despite the introduced perturbation, single-mode lasing and its spatial profile remain in the whole range of pump power (up to 2.4 GW m^−2^ that is ~3 times the lasing threshold) used in our experiment. This robustness arises from the topological robustness of the defect even with nonlinearity above the threshold (see Supplementary Note 2)

One of the most important features of topological states is their robustness against defects and disorder. In particular, the spectral features of the zero mode associated with the passive SSH model are known to be insensitive to off-diagonal perturbations represented by the coupling coefficients. In active SSH systems, however, we find that the system can still display a certain level of immunity against diagonal perturbations. In other words, the zero-mode lasing can well survive with onsite perturbations despite a slight spectral shift of the mode energy (see Supplementary Note 4 for a quantitative analysis). This behaviour is exemplified in Fig. 4a, illustrating the modelled spectrum, and in Fig. 4b, showing the modified zero-mode profile when the resonant frequency of the third microring from the right is perturbed (as schematically labelled in the inset of Fig. 4a). To confirm these predictions experimentally, we introduced a polymer layer on top of the third ring resonator from the right to introduce a shift in its resonant frequency (see the SEM image in Fig. 4c) and measured the emission spectrum (Fig. 4d) and the lasing mode profile (Fig. 4e). It is evident that the zero-mode lasing still persists with a high extinction ratio, without appreciable change in the spatial emission profile apart from very small intensities leaking to the otherwise dark sublattice.

## Discussion

We have presented the first demonstration of a topologically robust single-mode hybrid silicon microlaser. Our work, complementing recent efforts^41,42^, shows that the interplay between topology and non-Hermitian symmetries equips the emerging topological zero mode with a distinct mode profile that enables it to fully exploit the distributed gain domains, while simultaneously spoiling other states through deliberately introduced optical absorption. The demonstrated laser action is stable and immune to moderate perturbations and defects since the zero mode is topologically protected by the applied symmetries. Compared to other mechanisms to obtain single-mode lasing, the present mechanism utilising topological features is unique in several respects: the lasing mode makes optimal use of the gain since the defect state resides only on the gain site, and displays a mode profile that is distinct from the extended states that one would obtain from other type of defects. The mode is protected against a large class of symmetry-preserving perturbations, and as it automatically sits in the centre of a spectral gap it is also more robust against symmetry-breaking perturbations (as are unavoidable in the experiment). Finally, the mechanism provides single-mode lasing for an arrayed supermode as opposed for the longitudinal mode of a single microring. Interestingly, as explained in Supplementary Note 2, the special features of the zero mode hold even when nonlinear gain saturation is taken into account. Realised in a hybrid III–V/silicon platform, the accomplished topological hybrid silicon microlaser supports large-area single-supermode operation, promising a highly efficient optical source for integrated silicon photonics to robustly feed power for chip-scale communication and computing.

## Methods

### Fabrication of the hybrid silicon laser

The topological microring laser was fabricated using direct bonding and overlay EBL processes. First, standard cleaning procedures were performed to prepare InGaAsP/InP and SOI wafers for the direct bonding, including acetone, isopropyl alcohol cleaning in a supersonic bath, and O_2_ plasma cleaning. Next, the InGaAsP/InP wafer was bonded with the SOI wafer, followed by a selective wet etching using HCl to remove only the InP substrate. Afterwards, a 500-nm-thick InGaAsP active layer was achieved on top of SOI. Then, the topological microring array was generated using EBL in hydrogen silsesquioxane, followed by chlorine-based dry etching through both InGaAsP and silicon layers. Another lithography step was then performed with accurate alignments, creating patterns in polymethyl methacrylate (PMMA) on every second microring, followed by deposition of Cr and lift-off of the sacrificial PMMA layer to realise the distributed gain and loss. We note that the etching process for forming the microring structure results in roughness on the sidewall, which in general favours the lasing of the TM modes over TE ones due to the reduced quality factor of the latter.

### Laser characterisation

We performed the characterisation of the lasing spectra and lasing intensity of the fabricated hybrid microring array based on optical pumping. The semiconductor laser was pumped by a nanosecond pulsed laser of a 50 kHz repetition rate and an 8 ns pulse duration at the wavelength of 1064 nm, which provides much stronger peak power to generate sufficient gain compared with a continuous wave laser and avoids heat accumulation due to its corresponding low duty cycle. To ensure uniform illumination of pumping on each of the nine microrings, a cylindrical lens and a Mitutoyo 20 × near-infrared (NIR) long-working distance objective (NA = 0.4) were utilised to span and focus the pumping beam along the array direction. The lasing emission from the fabricated laser array was collected by the 4-F system composed by the same NIR objective and a lens with a 20-cm focal length. Controlled by a flip mirror, the lasing field can be switched to the monochromator for characterisation of the spectral information, or to the infrared charge-coupled device camera for characterisation of the spatial intensity distribution with the pumping beam eliminated by a long-pass filter (1400 nm cutoff wavelength).

### Data availability

The datasets within the article and supplementary information in the current study are available from the authors upon request.

## Electronic supplementary material

Supplementary Information
