# Supplementary material for: Topological hybrid silicon microlasers
Source: Nat Commun. 2018 Mar 7;9:981. doi: 10.1038/s41467-018-03434-2 (PMC5841408; doi:10.1038/s41467-018-03434-2)
Supplement: Supplementary file 1 — Supplementary Information [file 41467_2018_3434_MOESM1_ESM.pdf]

Supplemental Information
Topological Hybrid Silicon Micolasers

Han Zhao[1], Pei Miao[2,3] Mohammad H. Teimourpour[4], Simon Malzard[5], Ramy El-Ganainy[4], Henning Schomerus[5] and Liang Feng[2*]

[1]*Department of Electrical and Systems Engineering, University of Pennsylvania, Philadelphia, PA 19104, USA*

[2]*Department of Materials Science and Engineering, University of Pennsylvania, Philadelphia, PA 19104, USA*

[3]*Department of Electrical Engineering, The State University of New York at Buffalo, Buffalo, NY 14260, USA*

[4]*Department of Physics and Henes Center for Quantum Phenomena, Michigan Technological University, Houghton, MI 49931, USA*

[5]*Department of Physics, Lancaster University, Lancaster, LA1 4YB, United Kingdom*

*Email: fenglia@seas.upenn.edu

Supplementary Note 1: Topological properties of the SSH chain with loss and gain. The notion of topology deals with how discrete features (such as a quantized phase) can change under smooth deformations. Expanding the parameter space by considering more complex systems having more additional degrees of freedom can either spoil the discreteness of the topological features or make different values effectively indistinguishable. It is thus not possible to make an intuitive leap by assuming that topological robustness persists when new ingredients are added to an otherwise topologically protected system. In our experiment, the topological array is, by definition of a laser, active and also nonlinear. This in turn necessitates to revisit the topological concepts in this system. Given these observations, we first recapitulate the topological characterization of the Su-Schrieffer-Heeger (SSH) model in the original Hermitian electronic setting[1-3] and then explain how this extends to non-Hermitian photonic systems with loss and gain[4].

<u>Standard (passive) scenario</u>

The original SSH model can be phrased as a set of coupled-mode equations,

$$i\frac{dA_n}{dt} = t_n' B_n + t_{n-1}'' B_{n-1}, \quad (1)$$

$$i\frac{dB_n}{dt} = t_n' A_n + t_n'' A_{n+1}, \quad (2)$$

where A_n and B_n represent amplitudes on the two sites in the nth dimer, and t_n' and t_n'' are the effective couplings within and to the next dimer, which we assume to be real and positive. In an infinitely periodic chain with fixed $t_n' = t'$ and $t_n' = t''$ (e.g. taking the values t_1 or t_2 from the experiment) we can separate out the periodic time and space dependence, $A_n = A\exp(-i\omega t + ikn)$, $B_n = B\exp(-i\omega t + ikn)$ which leads to a dispersion of two symmetric bands $\omega = \pm\sqrt{t'^2 + t''^2 + 2t't''\cos k}$ that are separate by a gap of size $\Delta = 2|t' - t''|$. The symmetry of the spectrum arises from the operation $A_n, B_n \to A_n, -B_n$, whose effect is equivalent to inverting all couplings and therefore changes the sign of the effective Hamiltonian H, with elements

$$H_{nm} = t_n'\delta_{n+1,m} + t_n'\delta_{n,m+1}, \text{ if } n \text{ is odd}, \quad (3)$$

$$H_{nm} = t_n''\delta_{n+1,m} + t_n''\delta_{n,m+1}, \text{ if } n \text{ is even.} \tag{4}$$

In other words, chiral symmetry implies the existence of an operator χ such that $\chi H \chi^{-1} = -H$,thus forcing the spectrum to be symmetric around the zero line. In our case, such an operator χ corresponds to a unitary transformation

$$\sigma_z H \sigma_z = -H \ , \tag{5}$$

where σ_z is a Pauli matrix in the space of A and B sites, and constitutes a chiral symmetry[1,3].

This spectral constraint goes along with a constraint of the wave functions: all extended states have equal amplitudes $|A| = |B|$ on both sublattices, which means that their intra-dimer polarization $P = |A|^2 - |B|^2$ vanishes. Therefore, $\frac{A}{B} = \frac{t'+t''\exp(ik)}{\omega} = \exp(i\varphi)$ defines a complex phase φ which depends on k. As one inspects the change of the phase through the Brillouin zone, one then encounters two scenarios: for $t' < t''$ this phase increases by 2π (winding number 1), while for $t' > t''$ the phase returns to 0 (winding number 0). The transition at $t' = t''$ between both cases coincides with the closing of the gap.

In a semi-infinite system $n \leq 0$ B with boundary condition $B_0 = 0$,this winding number determines the existence of edge states[1]. This follows from the behaviour of the complex reflection coefficient $r = \frac{A+iB}{B+iA}$ at energies within the gap, where one encounters evanescent modes with $k = i\kappa$ that decay away from the interface. The winding numbers of the bulk solutions fix the reflection coefficient at the band edges to symmetry-protected values; for $t' < t''$ the coefficient r winds from 1 at the lower edge of the gap to -1 at the upper edge, while for $t' > t''$ the winding proceeds from -1 to 1.Causality implies that between these values, the reflection coefficient monotonously turns into the counter-clockwise direction, so that only in the case $t' > t''$ it passes through the boundary condition $r = -i$. Spectral symmetry dictates that this solution sits at $\omega = 0$.Note that this zero-mode has a finite polarisation $P = 1$. Furthermore, perturbations of the couplings do not affect the chiral symmetry and do not change the winding numbers, given that the start and end points are protected by symmetry. The zero-mode therefore exists even when the system is not exactly periodic. Indeed, it also exists when the hard termination is replaced by a weak

coupling to another SSH chain supporting the decay into the opposite direction (hence a different winding number), which is equivalent by reflection symmetry and results in the interface defect state utilized in the experiment.

Non-Hermitian case

The presence of gain and loss modifies the coupled-mode equations so that they read

$$i\frac{dA_n}{dt} = t_n' B_n + t_{n-1}'' B_{n-1} + i g_n^{\mathrm{A}} A_n, \quad (6)$$

$$i\frac{dB_n}{dt} = t_n' A_n + t_{n-1}'' A_{n+1} + i g_n^{\mathrm{B}} B_n, \quad (7)$$

where g_n^{A}, g_n^{B} describe the gain (loss if negative). The effective Hamiltonian now also contains complex diagonal elements

$$H_{nn} = i g_n^{\mathrm{A}}, \text{ if } n \text{ is odd}, \quad (8)$$

$$H_{nn} = i g_n^{\mathrm{B}}, \text{ if } n \text{ is even}, \quad (9)$$

which render it non-Hermitian. These terms change sign if one includes a complex conjugation, that is, an operator η such that $\eta H \eta = -H^*$. In the non-Hermitian case described by Supplementary Equations (6) and (7), such an operator η also corresponds to the Pauli matrix σ_z in the space of A and B sites, leading to the notion of a non-Hermitian charge-conjugation symmetry

$$\sigma_z H \sigma_z = -H^*, \quad (10)$$

which constraints the spectrum to be symmetric about the imaginary axis[4,5]. In an infinitely periodic chain the system still possesses two bands, which remain real as long as $|t'-t''| > |g^{\mathrm{A}} - g^{\mathrm{B}}|$.As before, all extended states still have equal amplitudes $|A| = |B|$ on both sublattices, so that we can once more inspect the winding of $A/B = \exp(i\varphi)$. Depending on the sign of $t'-t''$,this results in the same winding numbers as before, which also fixes the trajectory of the reflection coefficient $r = \frac{A+iB}{B+iA}\cdot\frac{|B+iA|}{|A+iB|}$ (now normalized to conserve the probability flux), and thus the existence of edge states. Besides a finite polarization, this mode now exhibits another distinct feature: its frequency is fixed to $\omega_0 = i g^{\mathrm{A}}$ (we assume the same termination site as before), i.e. it sits on the symmetry-protected

imaginary axis, while all extended states obey $\mathrm{Im}\{\omega\} = (g^{\mathrm{A}} + g^{\mathrm{B}})/2$. This difference in life time is the key to the topological mode selection, as the zero mode is favoured if $g^{\mathrm{A}} > g^{\mathrm{B}}$.

Supplementary Note 2: Nonlinear effects of lasing at the topological state in SSH chain. Although it is customary to make linear approximations when discussing laser characteristics near lasing threshold, the stabilization of the laser operation (without unbounded growth) can be explained only when nonlinear interactions are accounted for. However, symmetry consideration under nonlinear conditions can be complex and subtle due to the rich effects brought by the breakdown of linear superposition. To establish the characteristics of the topological laser device demonstrated in this work, particularly those pertinent to the mode competition, we generalize the gain coefficients to include gain saturation nonlinearity[6]: $g_n^{\mathrm{A}} \to g_n^{\mathrm{A}}/(1+|A_n|^2)$, $g_n^{\mathrm{B}} \to g_n^{\mathrm{B}}/(1+|B_n|^2)$. Interestingly, even in this case, we can formulate a more general charge-conjugation symmetry as a constraint on the full dynamical evolution: for any solution $A_n(t)$, $B_n(t)$ there exists a partner solution $A_n^*(t)$, $-B_n^*(t)$. Stationary states still exhibit a harmonic time-dependence $\exp(-i\omega t)$, where ω is now real to signify stabilization at an overall balance between gain and loss. In this setting, the zero-modes now emerge as self-symmetric stationary states, which then automatically obey $\omega_0 = 0$. Therefore, the notion of topological protection also persists in the nonlinear case. This observation also opens up the possibility of novel phase transitions, due to spontaneous symmetry-breaking and time-dependent states[7,8], which in microlasers are expected to set in at large gain and are subject to further investigation.

Supplementary Note 3: Mode competition in topological microring lasers with multiple transverse modes. The microring resonators used in our experiment have a relatively large waveguide cross section with a width of 1 µm (inner and outer ring radii of 3.5 µm and 4.5 µm) and a height of 720 nm (a 500 nm thick layer of InGaAsP on top of a 220 nm layer of silicon). This design has two advantages: (1) it increases the lasing efficiency by providing a large spatial overlap between the lasing mode and the gain material; and (2) it results in bandwidth broadening in the case of a uniform pumping scheme (before adding the Cr layer), thus providing us with a spectral fingerprint to clearly differentiate between the lasing in the topological and non-topological modes.

Supplementary Figure 1a depicts four of the several different TM modes supported by the ring structure, including the fundamental TM mode (here labelled as TM_{11} mode) and three higher order modes (TM_{12}, TM_{21} and TM_{13} modes). The field profile of the higher order modes is less confined inside the ring and thus provides a stronger coupling to the corresponding modes in the adjacent ring. As a result, when the Cr layer is added, higher order modes exhibit more loss, or equivalently smaller quality factors. Therefore, although every set of transverse modes can form their respective topological states, the deposition of loss spoils the modes with higher coupling coefficients, favouring the selection of the fundamental TM_{11} mode in the lasing spectrum.

This intuition is confirmed in Supplementary Figure 1b, which depicts the dispersion of the normalized complex eigenenergies. Clearly, the topological state made of the fundamental TM_{11} mode, which has the smallest coupling coefficients, remains at zero energy and exhibits the least loss (smaller imaginary part of the eigen frequency), consequently containing the highest quality factor. For completeness, Supplementary Figures 1c-e show plots of the topological states corresponding to the TM_{11}, TM_{12} and TM_{21} modes. As expected, the field profile of the TM_{12} and TM_{21} modes hybridizes more strongly with the extended multimode states than for the TM_{11} mode. Because of the enhanced hybridization, the topological states of the TM_{12} and TM_{21} modes extend into the lossy sites (having the Cr layer). As a result, the topological state of the TM_{11} mode has the largest spatial overlap with the gain region made from the InGaAsP multiple quantum wells. On the other hand, in the absence of the lossy Cr layer and under uniform pumping of the full array, all the TM modes experience the same gain and contribute to the laser emission, which explains the observed multiple peaks in the broad emission spectrum of Fig. 3a in the main text.

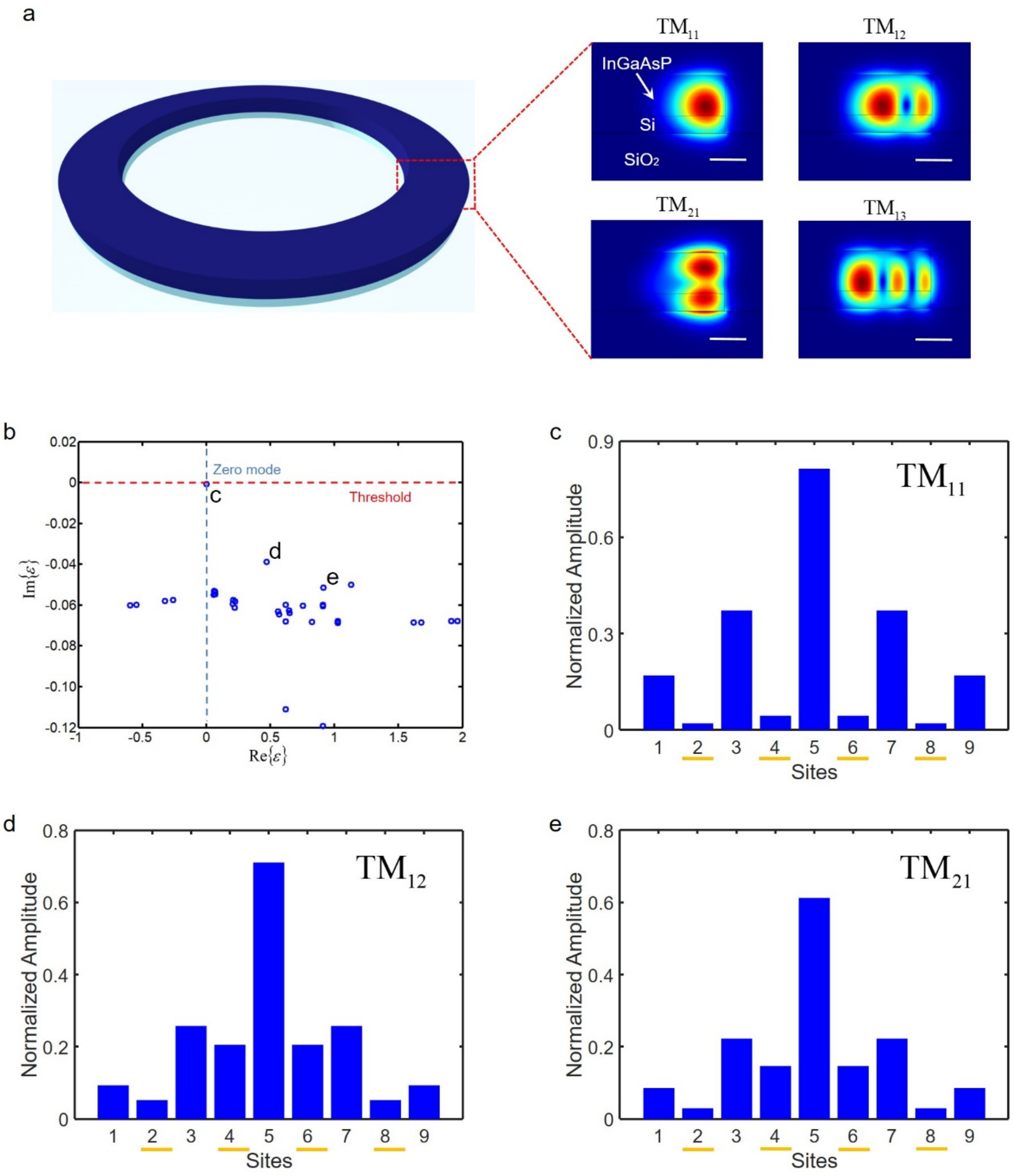

Supplementary Figure 1. Mode selection in a multi-mode topological microring laser. a The large cross section of the microring structure can support multiple transverse modes with a high quality factor, such as TM_{11}, TM_{12}, TM_{21}, and TM_{13}, which all participate in the mode competition process. Scale bars in the pseudo-coloured mode profiles: 400 nm. **b** The dispersion of the complex eigenenergies of the multi-mode tight-binding model shows the topological state of the fundamental TM_{11} mode having the smallest lasing threshold. The field distributions of eigenstates labelled by c, d, e are shown in panels **c-e**, corresponding to the topological states of TM_{11}, TM_{12}, and TM_{21}, respectively. Importantly, the lasing topological state of the fundamental TM_{11} mode shown in **c** almost stays unchanged in the presence of multi-mode couplings, while

the field profiles of the higher order modes TM_{12} and TM_{21} diffuse into the lossy sites (labelled in yellow marks) because of their stronger couplings. For even higher order modes, the coupling becomes stronger. This scheme thus naturally favours the lasing of the topological state made of the TM_{11} mode. In calculating these modes, the cross couplings between different mode families (i.e. TM_{11}, TM_{12}, TM_{21}) are also accounted for.

To further elucidate this mechanism of the multi-transverse mode hybridizations as another dimension of mode selection of the fundamental TM_{11} topological state in the devised microlaser array, we plot in detail the distributions of the TM_{11}, TM_{12} and TM_{21} topological states in each transverse supermode of the ring array, respectively in Supplementary Figures 2-4. Except for the fundamental mode TM_{11}, all other high-order transverse topological states strongly couple with other transverse orders and then form hybrid supermodes. Such mode hybridizations grow due to the large coupling coefficients between the higher order transverse modes, which drags their corresponding topological states into the lossy sublattice and consequently spoil the quality factors.

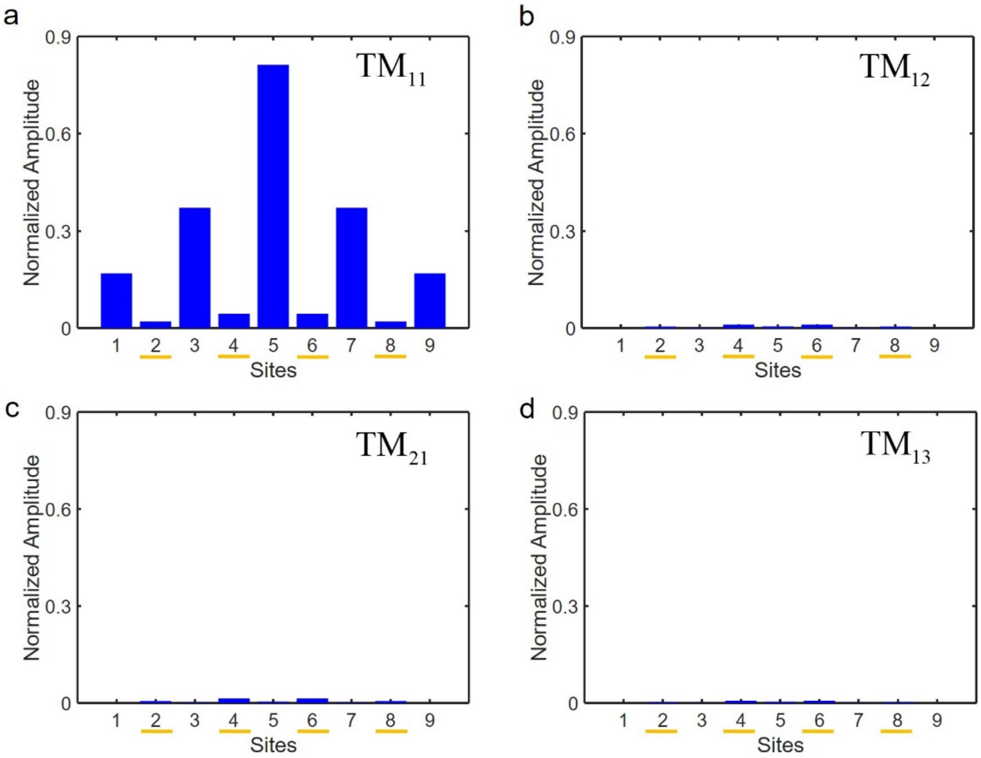

Supplementary Figure 2. Topological state of fundamental TM_{11} mode in presence of inter transverse mode couplings. a The field amplitude distribution on the TM_{11} mode. **b** The field amplitude distribution on the TM_{12} mode. **c** The field amplitude distribution on the TM_{21} mode. **d** The field amplitude distribution on the TM_{13} mode. Due to high quality factor of TM_{11} ring-resonance mode, the interactions between the

topological mode with other higher order extended modes are minimum, which preserves the intra-dimer polarisation on the gain sites and pins the lasing wavelength at zero energy (the resonance of TM_{11} mode in individual gain site). The yellow marks indicate the sites with loss.

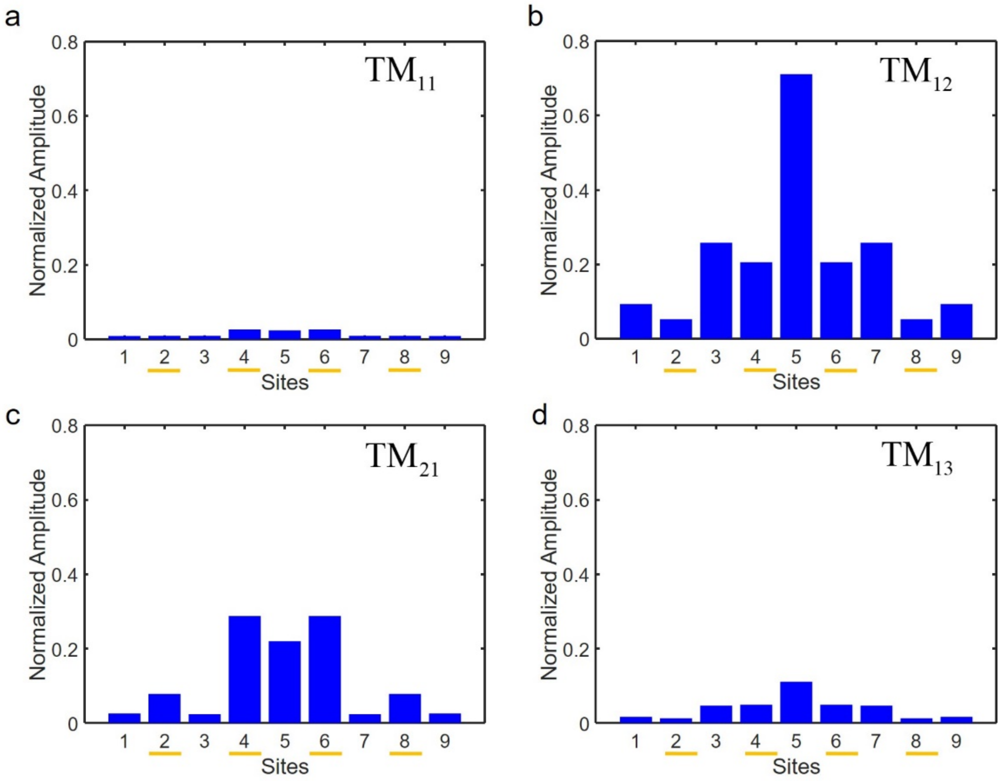

Supplementary Figure 3. Topological state of TM_{12} mode in presence of inter transverse mode couplings. a The field amplitude distribution on the TM_{11} mode. **b** The field amplitude distribution on the TM_{12} mode. **c** The field amplitude distribution on the TM_{21} mode. **d** The field amplitude distribution on the TM_{13} mode. While the field dominantly locates on TM_{12} mode of each ring sites, the non-vanishing couplings with TM_{21} and TM_{13} modes provide hybridizations with the extended supermodes, which drag the field into the lossy ring sites (yellow marks) and spoil the topological state from lasing.

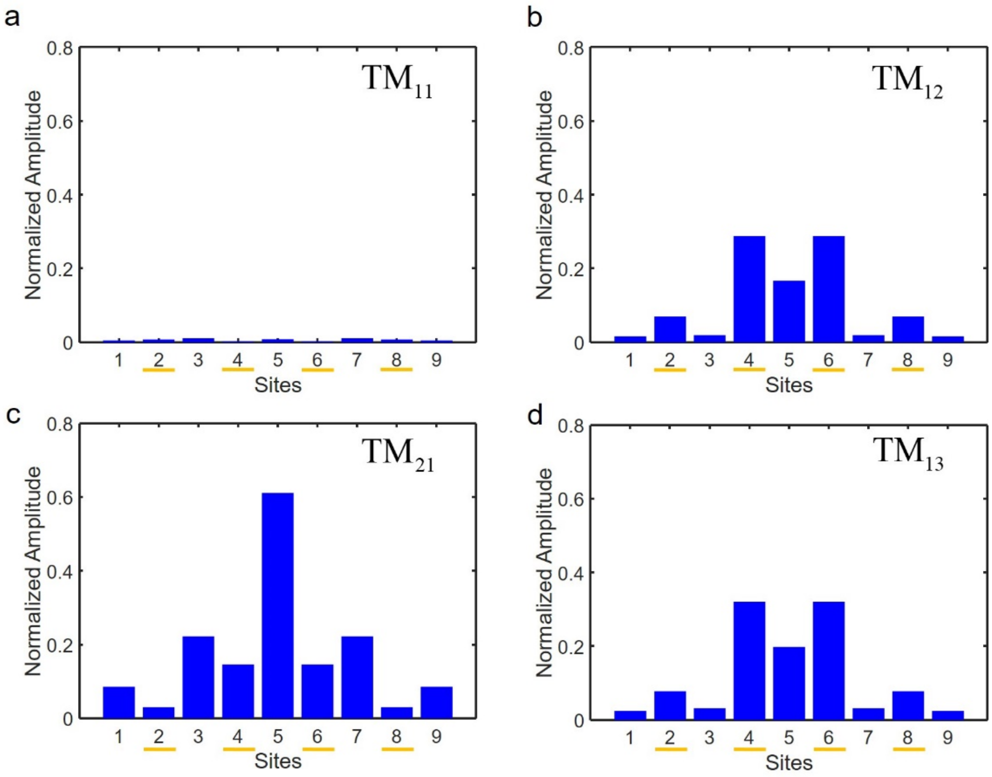

Supplementary Figure 4. Topological state of TM_{21} mode in presence of inter transverse mode couplings. a The field amplitude distribution on the TM_{11} mode. **b** The field amplitude distribution on the TM_{12} mode. **c** The field amplitude distribution on the TM_{21} mode. **d** The field amplitude distribution on the TM_{13} mode. Similar to the topolgical state of TM_{12} mode, the non-vanishing couplings with the other transverse modes deteriorate the intra-dimer polarisation and lower the quality factor of this topological state. The yellow bars indicate the sites with loss.

We note, however, if the cross couplings between different transverse orders in two adjacent rings are neglected, different transverse orders would possess their SSH Hamiltonians independently and thus formulate the topological states similar to the topological state from the fundamental TM_{11} mode, which result in several sublattice polarized supermodes with eigenenergies corresponding to their respective onsite resonances (zero energies) of these higher order transverse modes. If this was the case, the quality factors of these topological states would not be depress by the presence of losses on each second ring because of the dominant intra-dimer polarization, potentially leading to multi-peak lasing spectrum inherited from the Hermitian counterpart (Fig. 3a of the main text). On the contrary, the observation of single-mode lasing attests the existence of the

substantial cross couplings which ensures the mode selection amongst all the topological states by the least mode hybridization of the TM_{11} topological state over the others.

Finally, we note that the free spectral range between the longitudinal modes is ~25 nm. This large spectral separation facilitates the longitudinal mode selectivity via the gain curve of the semiconductor laser itself. In our case, the lasing modes have the azimuthal quantum number $m = 54$.

Supplementary Note 4: Onsite perturbation by PMMA deposition on the third ring from the right. In our experiments, the onsite perturbation is deliberately introduced by the deposition of a thick polymer layer on top of the third ring from the right. The consequent frequency shift of the single-ring resonance is estimated to be 212 GHz (corresponding to 2 nm wavelength shift), which is at the same level of the coupling strength at 200 nm – 300 nm separations. As we show in Fig. 4 in the manuscript, a moderate perturbation strength equivalent to the coupling strength only slightly modifies the field profile around the perturbed site, and the single-lasing behavior still survives.

In principle, this onsite perturbation effectively adds an energy offset on the corresponding diagonal entry of the SSH Hamiltonian, and breaks the charge-conjugation symmetry which allows the topological state to drift away from the zero energy. Indeed, if the perturbation strength is much larger, the topological state will be strongly altered and even disappear from the lasing spectrum due to the mode competition with a trivial defect state induced by the onsite perturbation. We plot in Supplementary Figure 5 and Supplementary Figure 6 the field profiles of the perturbed topological state and the induced trivial defect state with offset energy equivalent to coupling strength and 7 times the coupling strength, respectively. While the single-mode selection of the topological state stays valid under weak onsite perturbation, the increasing perturbation amplitude localizes the trivial defect state on the perturbed gain site and further hybridizes the topological state with the lossy sublattice, eventually leading to an even larger lasing threshold of the topological state compared to that of the trivial defect state. Therefore, a large onsite perturbation can possibly destroy the topological state and induce strong lasing behavior from the trivial defect state. However, we emphasize such breakdown of mode selection of the topological state requires significant onsite resonance disorder corresponding to more

than 10 nm shift of the onsite resonance wavelength. Therefore, we claim the topological lasing state can still survive moderate diagonal perturbations.

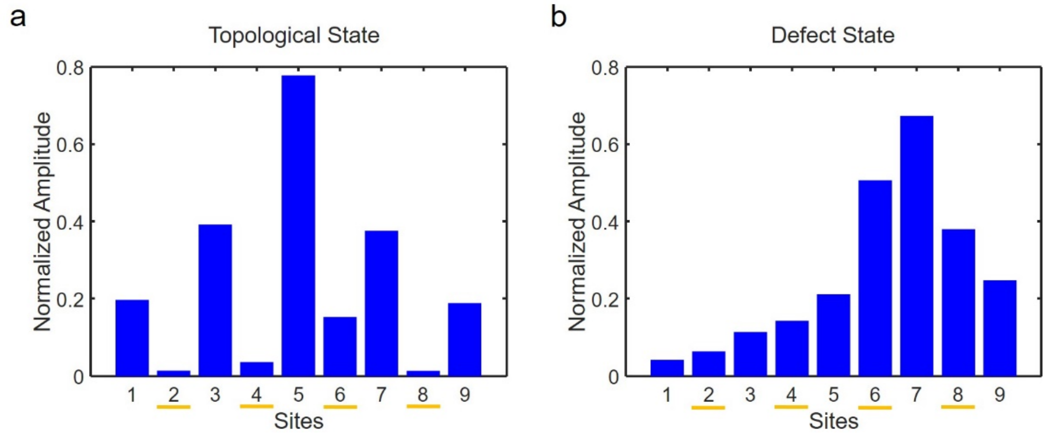

Supplementary Figure 5. Normalized mode profiles of the topological state and the defect state under perturbation equivalent to the coupling strength. a Field profile of the perturbed topological state. **b** Field profile of the induced trivial defect state. Both eigenstates are self-normalized such that the norm of the mode amplitude is identity. The weak onsite energy offset maintains the single-mode selection of the topological state due to the dominant field distribution on the gain sites, while the trivial state is well-suppressed because of a wide diffusion through the whole lattice. The yellow marks indicate the lossy ring sites.

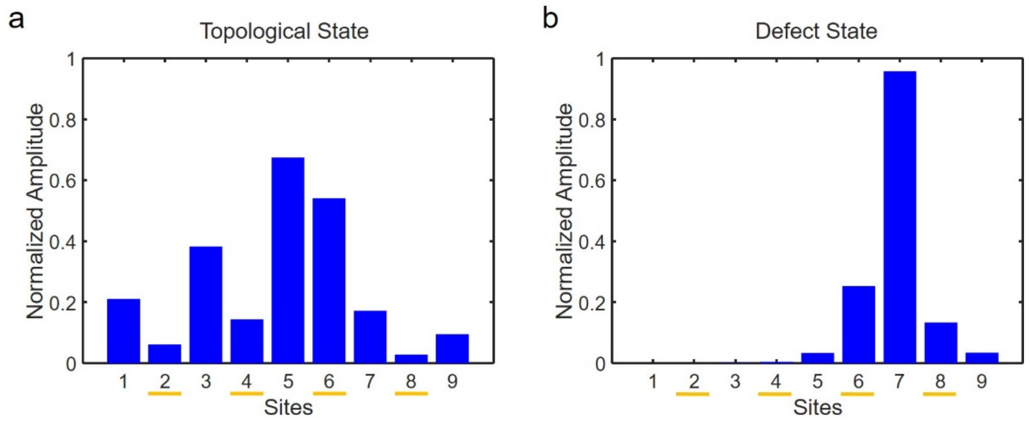

Supplementary Figure 6. Normalized mode profiles of the topological state and the defect state under perturbation equivalent to 7 times the coupling strength. a Field profile of the perturbed topological state. **b** Field profile of the induced trivial defect state. Due to an enhanced localization on the perturbed gain site, the trivial defect state possesses significantly reduced lasing threshold, while the topological state is spoiled by the hybridization with the lossy sublattices (labelled in yellow marks). Thereby, a lasing mode switching might be expected with significant diagonal perturbations.

Finally, we note that, in our structure without deliberately introduced onsite energy perturbation, the left and right halves of the microlaser array are in fact symmetric with respect to the central defect site. What matters is that they are both topologically distinct - it then depends on the definition of the unit cell to decide which one is termed trivial and which one is termed nontrivial[9-11]. Therefore, the robustness is independent of whether the

perturbed site is on right or left half of the array. Without loss of generosity, we introduced the onsite perturbation on the right side of the structure.

Supplementary References

1. Asbóth, J. K., Oroszlány, L. & Pályi, A. *A short course on topological insulators: band structure and edge states in one and two dimensions*. (Springer, 2016).
2. Su, W. P., Schrieffer, J. R. & Heeger, A. J. Solitons in polyacetylene. *Phys. Rev. Lett.* **42,** 1698–1701 (1979).
3. Ryu, S. & Hatsugai, Y. Topological origin of zero-energy edge states in particle-hole symmetric systems. *Phys. Rev. Lett.* **89,** 077802 (2002).
4. Schomerus, H. Topologically protected midgap states in complex photonic lattices. *Opt. Lett.* **38,** 1912-1914 (2013).
5. Pikulin, D. I. & Nazarov, Y. V. Two types of topological transitions in finite Majorana wires. *Phys. Rev. B* **87,** 235421 (2013).
6. Malzard, S., Cancellieri, E. & Schomerus, H. Topological phases in nonlinear complex-wave equations with a time-preserving symmetry. Preprint at https://arxiv.org/abs/1705.06895 (2017).
7. Harayama, T., Fukushima, T., Sunada, S. & Ikeda, K. S. Asymmetric stationary lasing patterns in 2D symmetric microcavities. *Phys. Rev. Lett.* **91,** 073903 (2003).
8. Harayama, T., T., Sunada, S. & Ikeda, K. S. Theory of two-dimensional microcavity lasers. *Phys. Rev. A* **72**, 013803 (2005).
9. Qi, X. & Zhang, S. Topological insulators and superconductors. *Rev. Mod. Phys.* **83**, 1057-1110 (2011).
10. King-Smith, R. D. & Vanderbilt, D. Theory of polarization of crystalline solids. *Phys. Rev. B* **47**, 1651-1654 (1993);
11. Atala, M., *et al*, Direct measurement of the Zak phase in topological Bloch bands. *Nat. Phys.* **9**, 795–800 (2013)
